# An Estimation of the Backscattering Strength of Artificial Bubbles Using an Acoustic Doppler Current Profiler

**DOI:** 10.3390/s22051812

**Published:** 2022-02-25

**Authors:** Ho Seuk Bae, Won-Ki Kim, Su-Uk Son, Woo-Shik Kim, Joung-Soo Park

**Affiliations:** Agency for Defense Development, Changwon 51678, Korea; belfre@add.re.kr (H.S.B.); suson@add.re.kr (S.-U.S.); kimws27@add.re.kr (W.-S.K.); js.park@add.re.kr (J.-S.P.)

**Keywords:** backscattering strength, acoustic Doppler current profiler, artificial bubble

## Abstract

Acoustic Doppler current profilers (ADCPs) were developed to acquire water current velocities, as well as depth-dependent echo intensities. As the backscattering strength of an underwater object can be estimated from the measured echo intensity, the ADCP can be used to estimate plankton populations and distributions. In this study, the backscattering strength of bubble clusters in a water tank was estimated using the commercial ADCP as a proof-of-concept. Specifically, the temporal variations in the backscattering strength and the duration of bubble existence were quantitatively evaluated. Additionally, the PDSL (population density spectrum level) and VF (void fraction) of the artificial bubbles were characterized based on the obtained distribution characteristics using a PDPA (phase Doppler particle analyzer).

## 1. Introduction

Bubbles in the water can be developed by natural factors such as wind currents, as well as artificial factors such as ship maneuvering [1]. Bubbles may negatively affect the accuracy of sonar equipment including acoustic sensors, as they severely attenuate sound propagation. However, given that strong attenuation can block acoustic wave propagation, this phenomenon could also serve as a barrier from self-noise and ambient noise. Therefore, to take advantage of the potential noise-reducing effects of bubble clusters, a quantitative analysis of the acoustic characteristics of bubble clusters is required.

Typical examples of the acoustic characteristics of bubbles are void fraction (VF), which means the ratio of the volume occupied by air in the total volume, attenuation coefficients, and backscattering strength, which refers to the intensity of the acoustic signals that are reflected back from the bubble. These acoustic properties are predominantly influenced by bubble distribution, such as the population density spectrum level (PDSL), which means the number of bubbles per unit volume by size, and temporal, spatial variation, and so on.

Several methods have been proposed to quantitatively measure the acoustic characteristics of bubbles in water, including VF and temporal and spatial variation analysis. Optical- or laser-based measurement methods are examples of non-acoustic approaches [2,3]. These measurements have some important limitations, including low sensitivity when the size of bubbles is not consistent or when the VF is low.

Acoustic transmission measurements generally entail the acquisition of an acoustic signal that propagates through the bubble cluster using one or several receivers to measure sound speed changes or the attenuation caused by the bubble cluster. From the measurement results, useful information about the bubble cluster such as the distribution of bubble size and VF can be estimated. Lamarre and Melville (1995) and Terrill and Melville (1997) calculated sound speed through a bubble cluster by dividing the travel time by the offset distance between the source and receiver [4,5]. Furthermore, Caruthers et al. (1999) and Medwin (1977) calculated the bubble size distribution by measuring the signal amplitude difference at the transmitter and receiver [6,7]. These acoustic transmission measurements have been widely applied to analyze the acoustic characteristics of bubble clusters. However, these approaches have not been widely applied in field or survey measurements due to spatial limitations, as the transmitter and receiver must be deployed on opposite sides of the bubble cluster to measure the sound propagation.

Acoustic scattering measurements, on the other hand, allow for the characterization of bubble clusters at long distances. In this method, an acoustic pulse directed toward the bubble cluster is backscattered by the bubble cluster. The characteristics of the bubble cluster can then be obtained using the received backscattered signal. Thorpe (1982) adapted an echo sounder as a measurement device [8]. Vagel and Farmer (1982) used a side-scan sonar [9], and Weber et al. (2005) applied a multibeam sonar for signal acquisition [10]. As mentioned above, this method allows for the long-distance characterization of bubble clusters, thus eliminating potential disturbances to the bubble cluster caused by equipment deployment, in addition to facilitating spatial distribution analysis. With the recent development of imaging sonar techniques, acoustic-based scattering measurement approaches have been increasingly developed and adopted.

Our study thus sought to measure the acoustic characteristics of bubble clusters using an acoustic Doppler current profiler (ADCP), which measures the sound speed as a function of depth using the frequency shift induced by the water current. ADCP systems were originally used to measure water currents for ocean surveys. This technology is also sometimes used to aid in the positioning of sensors in mooring systems to compensate for current-induced drifting. The ADCP has been widely used in studies for estimating the scattering strength with a layer scatterer because it can simultaneously acquire the echo intensity of each layer with the current velocity measurement. Teledyne RD Instruments, a major manufacturer of ADCPs, also developed techniques for the estimation of the scattering strength with a layer scatterer [11,12], and many researchers have estimated the scattering strength of each layer using the echo intensity obtained with ADCP systems [13,14,15]. In particular, the characterization of the biomass, size distributions, and spatiotemporal distributions of zooplankton, a major volume scatterer in the ocean, has been actively conducted.

In this study, the acoustic characteristics including the backscattering strength at the boundary of the bubble layer and temporal variabilities were analyzed using the ADCP. This approach has significant advantages in terms of echo intensity because the impedance of the bubble layer is markedly distinct from that of the water layer. Section 2 briefly describes the methods used to obtain the backscattering strength. Furthermore, the design of the water tank experiment to measure the acoustic characteristics is summarized in Section 3. In Section 4, we quantified the backscattering strength and the duration of bubble existence in the water tank experiment and inferred the PDSL and VF of the bubble cluster using the bubble size distribution obtained using a PDPA (phase Doppler particle analyzer). Finally, we summarize and discuss our research results in Section 5.

## 2. Estimation of Backscattering Strength 

An ADCP is used to measure water currents using the acoustic signal. In general, the ADCP measures the Doppler shift from the moving particles in the water column [16]. Fortunately, this instrument can acquire the depth-dependent echo intensities at the same time. To obtain the backscattering strength using the depth-dependent echo intensities measured with the ADCP, an equation provided by the manufacturer was applied [12]. The backscattered volume scattering strength $S_{v}$ can be obtained as follows:

(1) $$S_{v}=C+10\log\left(\left(T_{x}+273.16\right)R^{2}\right)-L_{DBM}-P_{DBW}+2\alpha R+10\log\left(10^{k_{c}\left(E-E_{r}\right)/10}-1\right)$$

where C is a constant that depends on each measuring equipment, $T_{x}$ is the water temperature (°C), R is the distance between the acoustic sensor and scatterers (m), $L_{DBM}$ is a term related to pulse length (dB), and $P_{DBW}$ represents the transmission output (dB). Furthermore, α is the acoustical absorption coefficient (dB/m), $k_{c}$ is the correction term to convert the measured amplitude counts on the ADCP to decibels (dB/count), E is the RSSI (returned signal strength indicator) for each beam (count), and $E_{r}$ is the RSSI for noisy environments without signal (count). Therefore, the backscattering strength can be very easily calculated using the above-described equation with the measured echo intensity by applying the equipment parameters summarized in Table 1.

However, there are many cases where the operator does not know the equipment parameters. To deal with this situation, we also tried to calculate the backscattering strength $S_{v}$ directly from the volume reverberation using the sonar equation [17].

(2) $$S_{v}=RL+2TL-SL-10\log V$$

(3) $$RL=10\log\left(10^{Ek_{c}/10}-10^{E_{r}k_{c}/10}\right),$$

(4) $$SL=170.8+10\log P+DI_{T},$$

where *RL* is the received level reflected from the bubble cluster, *SL* is the source level, and 10log*V* is the volume steering by the transmitted beam. *TL* is the transmission loss containing the absorption. This varies according to the marine environment, so there are several formulas to define it. Teledyne RD Instruments suggested 2TL=20logR+2αR [11,12]. Flagg and Smith (1989) and Kang et al. (1994) suggested $2TL=20\log R+2\alpha R-10\log\left(10^{-3}D\right)$ [13,14]. Instead, Jurng (1996) applied $2TL=40\log R+2\alpha R-10\log\left(10^{-3}D\right)$ as a TL formula for the Korean peninsular environment [15]. In this paper, we followed Jurng (1996)’s approach [15]. *D* is the bin size for the ADCP. *P* represents the output voltage (115 W) and $DI_{T}$ represents the transmission directivity index (19.32 dB). V is the geometrically calculated steering volume induced by the beam, which is obtained by applying 2.2° of a 3 dB beamwidth and 0.9 m of pulse length. Finally, the volume scattering strength for the bubble can be estimated using the depth-dependent echo intensity acquired by the ADCP and the above-described sonar equation. 

## 3. Water Tank Experiment Design

To measure the backscattering strength at the bubble interface, an observation experiment was conducted in a water tank to eliminate the effects of real ocean environments such as wind and waves on the water surface. The experimental water tank, which was specifically designed to measure acoustical characteristics, was 20 m long, 10 m wide, and 10 m deep. To minimize specular reflection at the bottom, the bottom of the water tank was sloped.

To obtain meaningful data from the ADCP, the selection of appropriate equipment with a reliable frequency to cover the water depth and the required resolution is critical. In this study, we selected the “Workhorse Sentinel 300” model produced by Teledyne RD Instruments (Poway, CA, USA), as this equipment can conduct broadband signal processing at the 307.2 kHz band and is widely used in Korea.

**Figure 1:**
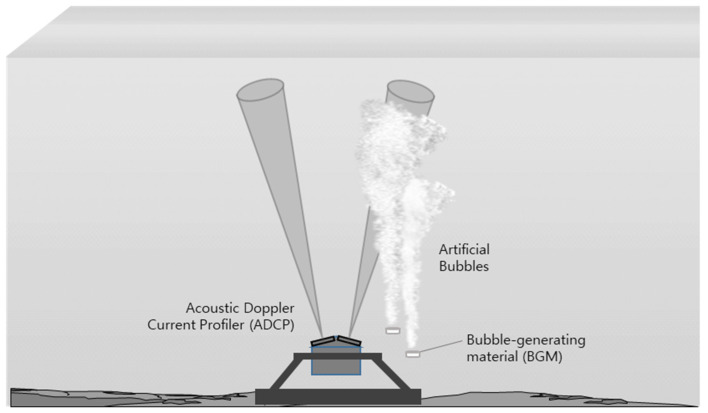
Schematic of the water tank experiment.

In this experiment, our ADCP was placed at the center of the water tank bottom using a fixable supplement device (gimbal) so that the sound beam could steer upward and remain horizontal. Furthermore, one beam (#3) was arranged to steer toward the direction in which the bubbles were located, and the other beams (#2 and #4) were intended to steer in a direction within the narrow width of the water tank. The remaining beam (#1) was directed toward the opposite direction of the bubble cluster. Figure 1 illustrates a diagram of our experimental setup.

In this experiment, we employed a bubble-generating material (BGM) that produces bubbles through chemical reactions when the material is immersed in water. The BGM is a pelletized material that sinks into the water and continuously generates artificial bubbles until the reaction stops.

To measure the acoustic properties of the artificial bubbles, we selected an acquisition bin size *D* (which is directly related to the spatial resolution of the ADCP) of 0.5 m, which is the minimum size that can be set by the equipment. Furthermore, the pulse repetition interval was set to 1 s. Among the setting parameters, there is an ensemble number related to the signal-to-noise ratio (SNR). The SNR increases with the number of pings required to obtain the ensemble average. However, given that the bubbles changed dynamically over time in our experiment, the application of an ensemble average using many pings negatively affected the superposition of the signal. Since the main purpose of our study was to observe the variation in the scattering strength of bubbles, we did not perform ensemble averaging (ping/ensemble = 1). 

The equipment can provide the acoustic velocity (mm/s), the correlation, the data fraction that passed specific criteria, and the echo intensity (count). In this experiment, the acoustic characteristics of the artificial bubbles were determined by acquiring the echo intensities of four transmitted beams to obtain the scattering strength of the bubbles. However, interference between the main lobe and the side lobe of the transmitted beam occurred near the water surface because our equipment with a 307 kHz transmission band had a tilted transmission angle of 20.0° from the water surface. Therefore, the signal within an area of approximately 6% near the surface was excluded. Furthermore, ringing occurred within approximately 2 m from the source. The signals affected by interference and ringing were not used in this study. Afterward, data were acquired using routine procedures for current velocity measurements using standard software.

## 4. Results

### 4.1. Estimation of the Backscattering Strength Using the Approach Provided by RD Instruments

**Figure 2:**
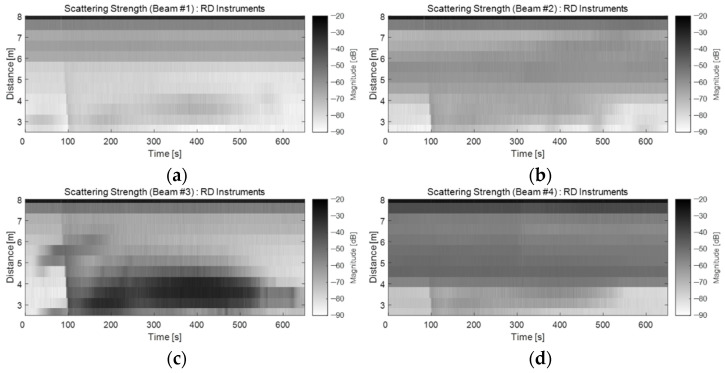
Backscattering strength $S_{v}$ derived from the method provided by RD Instruments: beams (**a**) #1, (**b**) #2, (**c**) #3, and (**d**) #4 of the ADCP.

Figure 2 shows the backscattering strength, $S_{v}$, derived from Equation (1), as suggested by the manufacturer (Teledyne RD Instruments, Poway, CA, USA). Figure 2a shows the results of the conversion of the signals obtained from beam #1 into scattering strength as two-dimensional images, thus facilitating the visualization of changes as a function of time and depth. Figure 2b–d show the results of converting beams #2, #3, and #4 into scattering strength, respectively. As illustrated in these figures, the echo intensity caused by the bubbles began to be strongly recorded as the BGM sank from the water surface to the bottom of the water tank (90 s). Based on these results, a BGM sedimentation velocity could be accurately estimated. In particular, the BGM was dropped toward the direction of beam #3 of our ADCP, and a strong echo intensity was identified at the bottom of the water tank after the BGM reached the bottom (Figure 2c). These results indicated that the BGM-generated artificial bubbles were continuously and strongly produced on the tank floor. However, because the acoustic beam transmitted from the ADCP was strongly attenuated as it passed through the bubble cluster, the acoustic level of the late arrival signal was inaccurate. Additionally, the bubbles could not be accurately characterized near the water surface.

In Figure 2b,d, the main lobe of the acoustic beam was directed towards the short width of the water tank. The reflected signal from the tank wall and the echo signal from the bubble cluster by the side lobe of the acoustic beam were recorded simultaneously from a distance of approximately 4 m.

**Figure 3:**
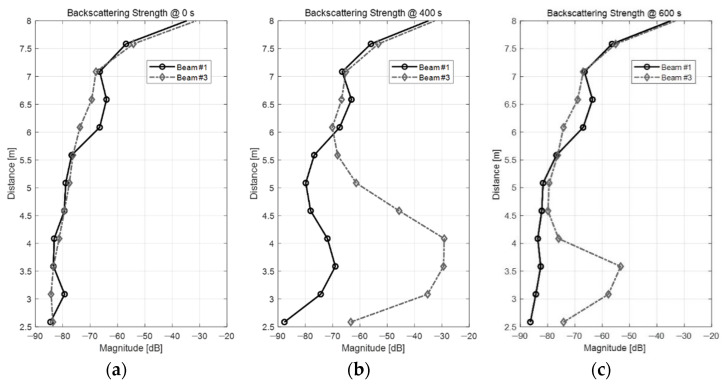
Backscattering strength profiles as a function of depth: (**a**) 0 s; (**b**) 400 s; and (**c**) 600 s.

To quantitatively determine the backscattering strength of the bubble cluster, we next analyzed the changes in the scattering strength as a function of depth and time. Figure 3 shows the scattering strength profiles using the data of acoustic beam #1 and #3 of the ADCP. Figure 3a illustrates a profile of the backscattering strength before the BGM was dropped on the water surface, which corresponds to 0 s in Figure 2. Figure 3b illustrates the profile at 400 s, when the BGM reacted most strongly. Figure 3c is the same profile at 600 s, when the bubbles began to dissipate. In Figure 3a, the backscattering strengths ranged approximately between −80 and −85 dB at a distance of 4 m in the water tank before the BGM was added. In contrast, Figure 3b shows that backscattering strength reached approximately −30 dB at a distance of 4 m once the BGM reacted with the water. This means that the artificial bubbles increased the backscattering strength by approximately 50 dB or more at a 4 m distance. These results confirmed the acoustic scattering properties of the artificial bubbles generated by the BGM developed herein. As shown in Figure 3c, the backscattering strength of the bubble cluster was substantially lower once the bubbles dissipated (Figure 3c) compared to the previous value (Figure 3b).

### 4.2. Backscattering Strength Derived Directly Using the Volume Reverberation Theory

**Figure 4:**
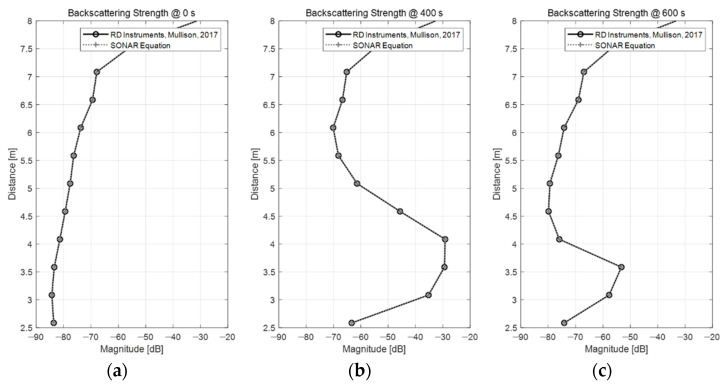
Comparison of backscattering strengths obtained through two different approaches: (**a**) 0 s; (**b**) 400 s; and (**c**) 600 s, respectively. The solid and dashed lines overlap almost perfectly in the figures.

The backscattering strength was calculated using Equation (2), which is based on the volume reverberation theory. Figure 4 compares the backscattering strength values derived from the previous section and the sonar equation (Equation (2)). 

In Figure 4, the black solid line represents the backscattering strength obtained with the method provided by the manufacturer, whereas the gray dashed line shows the results obtained using the sonar equation. In the figure, the solid and dashed lines overlap almost perfectly. This means that the two methods rendered very similar results, as expected.

In fact, the method suggested by the manufacturer is an equation derived from the sonar equation from an SNR perspective, and the device design variables are all considered in the equation through the *C* constant. Therefore, when the parameters are applied according to the ADCP specifications, the backscattering strength can be obtained with a very high accuracy. However, since the transmission loss model is fixed, environmental variability cannot be accounted for. On the other hand, the method derived from the sonar equation by the volume reverberation theory has a strong advantage in terms of applicability in marine environments because the effect of the ocean environment can be flexibly considered in the transmission loss model. Additionally, accurately designing parameters for ADCP measurement devices is not an easy task. However, if the detailed terms of the sonar equation are defined through measurements, accurate results can be obtained.

### 4.3. Duration of the Existence of Artificial Bubbles 

**Figure 5:**
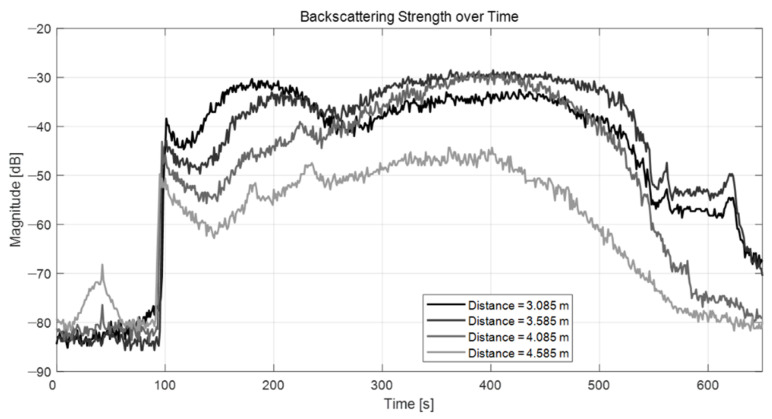
Backscattering strength of artificial bubbles over time.

By monitoring the level of the backscattering strength measured using the ADCP after BGM deployment, the duration of the existence of the artificially generated bubbles could be accurately estimated. Due to the different sizes and distributions of the bubbles along the water column, changes in the backscattering strength were characterized as a function of distance from the ADCP. Figure 5 shows the results of the backscattering strength level at different distances from the ADCP (approximately 3.0, 3.5, 4.0, and 4.5 m). Upon adding the BGM to the water, the backscattering strength values increased relatively quickly closer to the water surface as the pellets began to sink (4.5 m distance, gray line). In contrast, few effects were observed closer to the bottom of the water tank (3.5 m distance, black line). Additionally, our experiments confirmed that it took approximately 500 s to return to the pre-BGM backscattering strength level. Therefore, we could easily estimate that the artificial bubbles would last more than 7 min in the water column when accounting for the time it takes for the BGM to react with water until the bubbles fully dissipate. Therefore, the backscattering strength was an effective indicator of the presence of artificial bubbles in the water column.

### 4.4. Inference of the Population Density Spectrum Level and the Void Fraction of Artificial Bubbles

**Figure 6:**
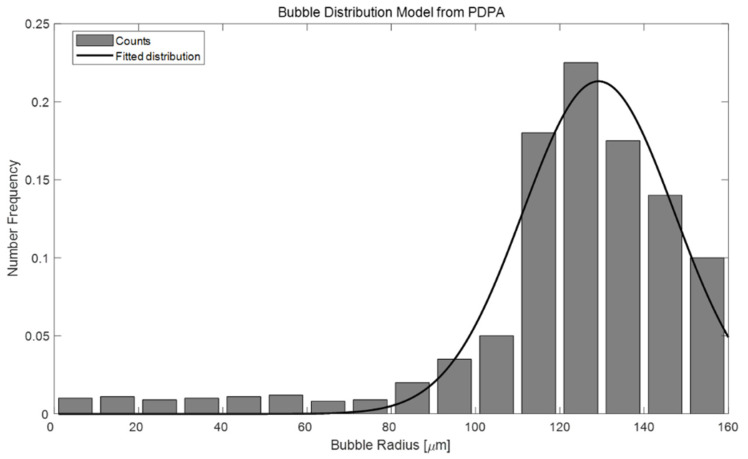
Artificial bubble distribution model measured by the PDPA.

In the previous section, the backscattering strength and existence duration of BGM-generated artificial bubbles were calculated using echo intensities measured with an ADCP device in an experimental water tank. The overarching goal of this study was to estimate the PDSL and VF of these artificial bubbles by measuring their backscattering strength. The PDSL and VF of the artificial bubbles are also essential acoustic properties for acoustic performance modeling. Here, the PDSL represents the proportion of each bubble size per unit volume. To estimate the PDSL, a distribution model must first be constructed according to the bubble size. Here, a PDPA measuring device was used to obtain this distribution model (Figure 6).

The PDPA can measure the bubble size, velocity, and population in the intersection of two focused laser beams. Bubbles yield light scattering of the two laser beams by creating optical interference patterns. A receiving detector converts the optical signal into a Doppler burst with a frequency linearly proportional to the velocity. We can obtain the bubble diameter from the phase shift information of the Doppler signals measured from different detectors [18].

To acquire the bubble distribution from the BGM, we set up a small-scale water-tank experiment with this PDPA instrument. The device was placed outside the water-tank, and two laser beams were set to cross a specific area through the transparent glass of the tank. After that, we recorded the PDPA data by dropping our BGM from the water surface to the bottom of the tank to pass through the intersection of laser beams.

The histogram in Figure 6 shows the cumulative bubble counts obtained through the PDPA, and the result of curve fitting is indicated by a solid black line. The distribution function was assumed to adhere to a Gaussian model and was calculated via the nonlinear least-squares method.

**Figure 7:**
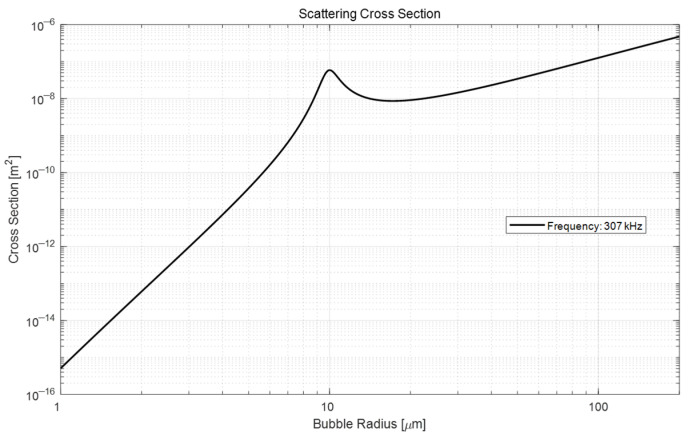
Scattering cross-section of the bubble size at the 307 kHz band.

The backscattering strength of the BGM-generated artificial bubbles was experimentally determined in a water tank. However, due to the dynamic changes in the distribution and sizes of the artificial bubbles in the water, the backscattering strength of the bubble cluster also changed as a function of water depth and time. Therefore, for convenience, the PDSL of the artificial bubbles was estimated by assuming a maximum backscattering strength of −30 dB. The scattering strength, $S_{v}$, can thus be expressed by Equation (5) as shown in the existing literature [19].

(5) $$S_{v}=10\log\left(\int_{0}^{\infty }\sigma_{s}n\left(a\right)da\right),$$

(6) $$\sigma_{s}=\frac{4\pi a^{2}}{{\left[{\left(f_{R}/f\right)}^{2}-1\right]}^{2}+\delta^{2}}$$

where a is the radius of the bubble and n is the bubble radius spectrum, which is the number of bubbles per unit volume per unit radius. In Equation (6), $\sigma_{s}$ is the scattering cross-section, and f and $f_{R}$ are the operation frequency and resonance frequency, respectively. δ is the damping constant, which accounts for viscosity, reradiation, and thermal conductivity. Figure 7 shows the scattering cross-section with each bubble size at the 307 kHz band (i.e., the operating frequency band of the ADCP equipment). Based on this figure, it can be estimated that there will be strong scattering due to the resonance at a radius of 10 μm.

Since we knew the bubble distribution curve (Figure 6) in advance, we could invert the number of bubbles per unit volume per unit radius *n*(*a*) by multiplying the bubble distribution curve by a proper constant to satisfying the scattering strength $S_{v}=-30\;\mathrm{dB}$ using Equation (5). 

**Figure 8:**
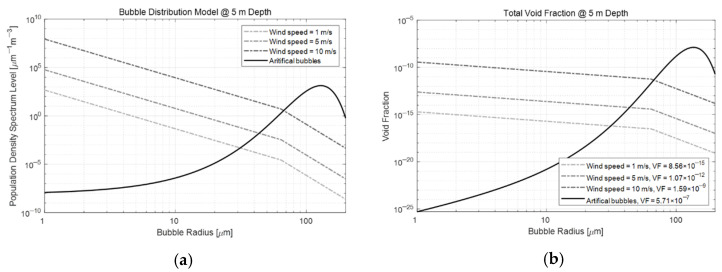
Estimated (**a**) PDSL and (**b**) VF of the artificial bubbles (solid lines) and natural bubbles (dashed lines).

The black solid line in Figure 8a represents the inverted *n(a)* model, PDSL. When the proportion of each bubble size was obtained, the VF could be calculated by multiplying the theoretical volume of the bubble (black solid line in Figure 8b). Our findings confirmed that the VF of the artificial bubbles had a value of approximately $5.71\times 10^{-7}$.

The PDSL and VF, which represent the distribution characteristics of the number of artificial bubbles by size, were estimated using the backscattering strength through the water tank experiment, as well as the distribution model by bubble size acquired through the PDPA device. To compare the magnitude of the estimated PDSL and VF distribution characteristics of the BGM-generated artificial bubbles, we showed them together with the PDSL and VF distribution characteristics of naturally occurring bubbles generated by natural wind in the ocean environment in Figure 8. The Hall–Novarini model was used to reproduce the spontaneous generation of bubbles in nature. This model is based on the PDSL model proposed by Hall and was partially supplemented by Novarini and Norton. However, several bubble generation models have been proposed (e.g., Vossen and Ainsline [20]). In this study, the naturally occurring bubble distribution model was implemented by applying the widely used Hall–Novarini model.

Using this model, we compared the characteristics of naturally generated bubbles at a water depth of 5 m when the wind speed was 1 m/s, 5 m/s, and 10 m/s. The dashed lines in Figure 8 represent (a) the PDSL and (b) VF characteristics of natural bubbles generated by wind. Based on these figures, we expected that only artificial bubbles with a radius of 30 μm or more would be observed when the wind speed was under 1 m/s. Additionally, we anticipated that artificial bubbles with a radius of less than 30 μm would not be easily observed compared to the naturally occurring bubbles. Similarly, we also knew that artificial bubbles with a radius of 70 μm or more at a wind speed of 10 m/s would be more easily observed. However, smaller bubbles would exhibit the same limitations as the <30-μm radius bubbles discussed above.

## 5. Conclusions

This study measured the acoustic properties of BGM-generated artificial bubbles using echo intensities at different depths obtained using an ADCP system. Our findings confirmed that the backscattering strength of the artificial bubbles generated by the BGM was up to −30 dB, and the duration of the existence of the bubbles in the water column exceeded 7 min. Additionally, we were able to derive the characteristics of the PDSL of the artificial bubbles using a distribution model for each bubble size measured with PDPA equipment. Furthermore, we successfully estimated that the VF of the bubble cluster was $5.71\times 10^{-7}$. By analyzing the characteristics of artificial (BGM-generated) and naturally occurring bubbles generated by wind currents, the radius of the observable artificial bubble could be inferred based on the wind speed.

It was quite challenging to measure the acoustic characteristics of artificial bubbles using the ADCP. Nevertheless, we consider that our method constituted a good first approach to effectively estimate the acoustic characteristics of artificial bubbles, as it uses commercial equipment that can be readily employed.

However, although the presence of artificial bubbles could be clearly identified through the echo intensity of the ADCP acoustic beam, additional studies are required to optimize the accuracy of this approach. Given that acoustic energy is very strongly attenuated by bubbles, the scattering strength derived from the late signal from the ADCP is considerably inaccurate. Furthermore, the limitations in the accuracy of the acquired echo intensity are significant because bubbles are dynamic objects and cannot be superposed on ensemble signals.

Nevertheless, the estimated acoustic characteristics of the artificial bubbles obtained using our approach can be suitably applied by our research team as input variables to develop analysis models for the evaluation of acoustical performance and effectiveness. In the near future, we plan to verify the reliability of the method proposed herein by analyzing measurements from real marine environments. 

## Figures and Tables

**Table 1:** Parameters for equipment setting.

*C*	*T_x_*	*L_DBM_*	*P_DBW_*	*α*	*k_c_*	*E_r_*
−140.87 dB	10.5 °C	−0.4576 dB	14.0 dB	0.025 dB/m	0.45 dB/count	40 count

## Data Availability

Not applicable.

## References

[1] Bae H.S., Kim W.-K., Son S.-U., Kim W.-S., Park J.-S. (2021). A Study on the Ship Wake Model under Ocean Environment. J. Korea Inst. Mil. Sci. Technol..

[2] Johnson B.D., Cooke R.C. (1979). Bubble populations and spectra in coastal waters: A photographic approach. J. Geophys. Res. Ocean.

[3] Stokes M.D., Deane G.B. (1999). A new optical instrument for the study of breaking waves at high void fractions within breaking waves. IEEE J. Ocean Eng..

[4] Lamarre E., Melville W.K. (1995). Instrumentation for the measurement of sound speed near the ocean surface. J. Atmos. Ocean Technol..

[5] Terrill E., Melville W.K. (1997). Sound-speed measurements in the surface-wave layer. J. Acoust. Soc. Am..

[6] Caruthers J.W., Stanic S.J., Elmore P.A., Goodman R.R. (1999). Acoustic attenuation in very shallow water due to the presence of bubbles in rip currents. J. Acoust. Soc. Am..

[7] Medwin H. (1977). In situ acoustic measurements of microbubbles at sea. J. Geophys. Res..

[8] Thorpe S.A. (1982). On the clouds of bubbles formed by breaking wind-waves in deep water and their role in air-sea gas transfer. Philos. Trans. R. Soc. London. Ser. A Math. Phys. Sci..

[9] Vagle S., Farmer D.M. (1992). The measurement of bubble-size distributions by acoustical backscatter. J. Atmos. Ocean Technol..

[10] Weber T.C., Lyons A.P., Bradley D.L. (2005). An estimate of the gas transfer rate from oceanic bubbles derived from multibeam sonar observations of a ship wake. J. Geophys. Res. Ocean.

[11] Deines K.L. (1999). Backscatter estimation using broadband acoustic Doppler current profilers. Proceedings of the IEEE Sixth Working Conference on Current Measurement (Cat. No. 99CH36331).

[12] Mullison J. Backscatter estimation using broadband acoustic Doppler current profilers-updated. Proceedings of the ASCE Hydraulic Measurements & Experimental Methods Conference.

[13] Flagg C.N., Smith S.L. (1989). On the use of the acoustic Doppler current profiler to measure zooplankton abundance. Deep Sea Res. Part A Oceanogr. Res. Pap..

[14] Kang D., Na J. (1994). Comparison of the Temperature Profile with the Backscattering Strength by the ADCP Data in the Southwestern Part of the East Sea. J. Korean Soc. Oceanogr..

[15] Jurng M.S. (1996). Estimation of Zooplankton Biomass by Volume Scattering Strength in Coastal Waters. Ph.D. Thesis.

[16] Christie D., Neill S. (2021). Measuring and Observing the Ocean Renewable Energy Resource. Reference Module in Earth Systems and Environmental Sciences.

[17] Urick R.J. (1983). Principles of Underwater Sound.

[18] Measurement Principles of PDA—Dantec Dynamics. https://www.dantecdynamics.com/solutions-applications/solutions/spray-and-particle/phase-doppler-anemometry-pda/measurement-principles-of-pda.

[19] Medwin H., Clay C.S. (1998). Fundamentals of Acoustical Oceanography.

[20] Vossen R., Ainslie M.A. (2003). The Effect of Wind-generated Bubbles on Sea-surface Backscattering at 940 kHz. J. Acoust. Soc. Am..

